# How Does Perceived Organizational Support Reduce the Effect of Working Environmental Risk on Occupational Strain? A Study of Chinese Geological Investigators

**DOI:** 10.3390/ijerph20010051

**Published:** 2022-12-21

**Authors:** Su Tao, Jinmiao Hao, Jicong Yu

**Affiliations:** 1 School of Marxism, China University of Geosciences (Beijing), Beijing 100083, China; 2 School of Economics and Management, China University of Geosciences (Beijing), Beijing 100083, China

**Keywords:** occupational strain, working environmental risks, risk perception, stress, perceived organizational support, socio-emotional support, instrumental support, geological investigators

## Abstract

Background: Sensitivity to working environmental risks is essential to ensure the safety of geological investigators, but persistent perceived risks may lead to occupational strain, with negative effects on physical and mental health. This study aimed to find ways to reduce the negative consequences of working environmental risk perception without losing situational awareness. Methods: A questionnaire survey was conducted with 268 participants from geological survey organizations, measuring their perception of working environmental risk, occupational strain, perceived organizational support, and other stressors. Results: (1) The perception of working environmental risk and occupational strain of geological investigators was significantly higher than that of administrative staff, managers, and scientific researchers. (2) Working environmental risk is an important predictor of occupational strain in geological investigators even after controlling for other stressors. (3) Different dimensions of perceived organizational support play different roles in stress management; socio-emotional support negatively predicts occupational strain, and instrumental support moderates the relationship between working environmental risk perception and occupational strain. Conclusions: Our findings identify working environmental risk as one of the most prominent stressors for geological investigators. Socio-emotional support directly reduces occupational strain, while instrumental support buffers the effect of risk perception on occupational strain.

## 1. Introduction

On 13 November 2021, four geological investigators from Yunnan, China, lost contact with their group and died after they entered the hinterland of Ailao Mountain for field work. This indicates that although the risk of geological field surveys is greatly reduced with the improvement of safety protection and emergency support, there are still potential risks. Risk perception plays an important role in safety decision making in a high-risk context [1]. Perceptions of risk lead to protective action [2] and effective risk communication strategy, which can prevent accidents in the workplace [3]. However, risk factors in the working environment have been regarded as a kind of work stressor [4,5]. Risks or accidents may lead to acute strain, consisting of varying degrees of emotional, psychological, and behavioral responses [6,7]. These short-term acute reactions are, in turn, presumed to have a negative impact on an individual’s long-term mental and physical health [8]. Meta-analyses have shown that work-related risk factors are significantly associated with common mental health problems, such as anxiety and depressive disorders [9,10]. Lee et al. also found that the ability to perceive environmental risk is related to emotion. Anxiety can help to prevent emergencies and increase alertness [11]. However, long-term anxiety is associated with job burnout [12], and thus, can potentially lead to psychological distress symptoms and impair an individual’s daily functioning [13]. Therefore, reducing the adverse effects of the perception of working environmental risk without losing situational awareness constitutes a major challenge.

There are different perspectives on how to deal with stressors in the workplace [14]. As early as 1967, Weiss proposed in his model that social support, individual personality, and organizational factors are the buffers of the relationship between stressors and stress response [15]. Firstly, geological investigators work in remote places with little contact with the outside world, such as mountainous areas. Usually, they cannot see their family or friends for several months, which not only makes it difficult for them to obtain social support from family and friends but also increases their strain due to work–family conflict [16]. So, it is difficult for geological investigators to obtain the social support needed to cope with stressors. Secondly, because personality traits are relatively stable, they are not discussed in-depth in this study. Overall, to reduce the negative impact of environmental risks of working on geological investigators, an organizational perspective is a better choice. There are many organizational factors that predict work outcomes, such as the organizational climate and organizational culture [17], organizational justice [18,19], organizational change [20], and organizational support [21,22,23]. Among these, perceived organizational support (POS) is most closely related to the performance and well-being of employees [22].

Perceived organizational support refers to organizational members feeling that they and their contributions are respected by the company. In short, it is the support employees feel they receive from the organization [23]. Since Eisenberger proposed the concept in 1986, researchers have assumed that perceived organizational support is a single dimension, on a continuum from low to high. However, some researchers have taken a different view, departing from the idea of a single-dimensional structure. For example, McMillan pointed out that single-dimensional perceived organizational support only considers intimacy and respect, ignoring instrumental support. Therefore, he proposed a functional model of social support [24,25]. The integrated model of organizational support includes socio-emotional support, which affects the quality of service delivery, and instrumental support, which affects the performance of core service tasks. The two dimensions are characterized by intimacy and esteem support, network integration (socio-emotional support), and information, material, and people support (instrumental support). Given that working environmental risk is an objective stressor, geological survey organizations have their own safety regulations; the training and implementation of these regulations provide more instrumental support than emotional support. Therefore, in this study, the functional model of social support is adopted and it is expected that these two types of organizational support will bring different results.

This study explores whether the working environmental risk and occupational strain of geological investigators are at a high level; whether working environmental risk has a prominent impact on their occupational strain; and whether the socio-emotional and instrumental support provided by the organization can alleviate the adverse effects of working environmental risks on geological investigators.

Firstly, we must clarify the level of working environmental risk and occupational strain of geological investigators. To solve this problem, administrative staff from the same organization were selected as the control group. Meanwhile, the questionnaires adopted in this study have multiple occupational norms in China. The working environmental risk and occupational strain of geological investigators can be compared with those of managers, researchers, and technical workers. Geological surveys require employees to work in extreme, isolated settings. Although field equipment has been improved, the conditions in some areas are still difficult to work and live in. Therefore, the perception of working environmental risk of geological investigators is expected to be higher than that of employees working in indoor settings. This high risk perception may lead to greater occupational strain. Based on this, we present

**Hypothesis** **1:** *The perception of working environmental risk and occupational strain of geological investigators is higher than that of administrative staff, managers, and scientific researchers.*

The second question to be answered in this study is whether working environmental risk is an important stressor for geological investigators. The working environmental risks discussed herein are not limited to specific events but rather include stressful circumstances or environmental conditions. Many studies have shown that environmental risks can cause stress for employees working in special environments, such as construction workers and electrical workers. The meta-analysis by Sverke et al. reported a negative correlation between job insecurity and performance (*ρ* = −0.20) [26]. Geological investigators are no exception. We expect that even with excluding the influence of other stressors, working environmental risk perception will also predict occupational strain. There is some debate about how stressors affect the stress response. According to the stress resource retention theory, stressors initiate the loss of coping resources. If an individual fails to block the process of loss of a resource or to obtain a compensation resource, the feeling of stress will increase with the persistence of stressors, resulting in a cumulative effect and forming a “loss vortex” [27]. From this point of view, the relationship between stressors and the stress response becomes closer with increasing working time. However, according to the adaptive model of stress, life events (including positive and negative events) will have an immediate impact on people’s emotions, cognition, and behavior. However, people will gradually adapt to these events; therefore, as the event continues, reactions will gradually return to the pre-event level. The relationship between a stressor and a worker’s response to it will gradually weaken and disappear [28]. For geological investigators, on one hand, the accumulation of resource loss may occur with longer employment; on the other hand, this may be accompanied by working experience, which can help workers cope with stressors. Therefore, this study does not make specific assumptions about the relationship between working years and stressors and strain. We propose

**Hypothesis** **2:** *Working environmental risk perception significantly predicts the occupational strain of geological investigators after controlling for other stressors.*

The third question answered in this study is whether the organization providing support can reduce the negative effects of working environmental risk. Past research has debated whether perceived organizational support acts as a moderator or mediator in stress. On the one hand, organizational support may be an independent variable, parallel to working environmental risk. For example, perceived organizational support can be a coping resource that predicts organizational outcomes such as improving employees’ well-being [29] and work engagement [30]; perceived organizational support can also be a mediator between stressors and strains [31]. On the other hand, there is debate on whether organizational support moderates the relationship between stressors and strains. For example, studies have shown that POS does not moderate any of the relationships between stressors and strains [31,32]. However, there are also studies that support the role of POS as a moderator between stress and the corresponding response [33,34]. This study aims to resolve this disagreement by dividing organizational support into socio-emotional support and instrumental support. Socio-emotional support refers to the organization helping individuals meet their interpersonal and belonging needs (such as intimacy, emotion, and care) through the establishment of relationships and respect. Socio-emotional support is a kind of social support for employees that manifests as the provision of resources to help workers cope with stress. It is not related to the working environmental risk; rather, it is more closely related to the interpersonal environment. We expect that perceived socio-emotional support is not a moderator between working environmental risk perception and occupational strain. Consequently, we propose

**Hypothesis** **3:** *The main effect of perceived socio-emotional support in predicting occupational strain is significant.*

Instrumental support refers to the organization helping individuals meet their self-actualization needs (such as achievement, power, influence, self-esteem, and autonomy) through institutions and guidance. A stressor is a double-edged sword [35,36]. High-risk situations for employees can lead to anxiety, threatening their well-being [11]; however, they may also be seen as a challenge driving their work motivation [37]. We hypothesized that whether environmental risk is seen as a threat or a challenge may be related to employees’ perception of their ability to deal with risks. Additionally, instrumental support provides essential help for employees to increase their confidence and efficacy in dealing with environmental risks. So, the risks can be seen as a challenge rather than a threat. Self-efficacy may also be a good resource for workers in coping with stressors [36]. Therefore, it is expected that the organization providing instrumental support may reduce the negative psychological outcomes caused by the working environmental risks. Consequently, we propose

**Hypothesis** **4:** *Perceived instrumental support moderates the relationship between working environmental risk perception and occupational strain.*

That is, working environmental risk perception has a weaker predictive effect on occupational strain in geological investigators with high perceived instrumental support compared to those with low perceived instrumental support.

## 2. Materials and Methods

### 2.1. Methodology

A questionnaire survey was adopted in this study. The survey was distributed among 20 organizations affiliated with the China Geological Survey (CGS) and the Chinese Academy of Geological Sciences (GAGS). The human resources commissioners of each organization selected a representative sample of the institution to which they distributed paper questionnaires or online questionnaires (for geological investigators who were in the field at that time). After giving informed consent, the participants answered all the questionnaires.

IBM SPSS Statistics and IBM AMOS (IBM Corp., Armonk, NY, USA) were used for data analysis. First, Harman’s single-factor test was used to test for common method bias [38]. Secondly, the means and standard deviations of working environmental risk perception and occupational strain of geological investigators were calculated. Independent-sample T-tests were used to compare the scores with those of the control groups to test Hypothesis 1. Thirdly, hierarchical multiple regression analysis was carried out for occupational strain. In Step 1, independent variables (working environmental risk perception) and control variables (demographic and other stressors) were included in the regression analysis to test Hypothesis 2. In Step 2, potential moderators (perceived socio-emotional support and instrumental support) were included in the regression analysis, and in Step 3, interactions between independent variables and potential moderators were included in the regression analysis to test Hypothesis 3 and Hypothesis 4 [39].

### 2.2. Participants

A total of 300 questionnaires were sent out, and 282 were returned. Furthermore, 14 participants with more than 3 items missing were excluded, and 268 participants remained. The participants were from six provinces, including Anhui, Guangdong, Hebei, Henan, Shandong, and Tianjin.

Among the participants, 210 were working as field geological investigators, 148 of which were male, 58 female, and 4 unspecified, with a mean age of 35.12 ± 9.61 and a mean of 12.19 ± 11.18 working years. For the field working time per year, 57 participants had less than 2 months, 71 participants had 3–5 months, 35 participants had 6–8 months, 45 participants had more than 8 months, and 2 were unspecified.

The participants also included 58 administrators from the same organization who did not work in the field, comprising 38 males, 19 females, and 1 unspecified, with a mean age of 39.25 ± 10.93 and a mean of 16.97 ± 12.70 working years.

### 2.3. Materials

Working Environmental Risk Perception: The *physical environment (PE)* dimension of the *Occupational Role Questionnaire (ORQ)* subscale from a Chinese version of the *Occupational Stress Inventory—Revised Edition (OSI-R)* was used to measure the perception of the working environmental risk of geological investigators [40,41]. The OSI-R is a package of questionnaires that systematically measures occupational stress, including occupational stressors, occupational strain, and coping resources. The reliability, content validity, construct validity, and predictive validity of the OSI have been tested in previous studies [40,41,42], and there are norms for many occupations in China [43]. The PE includes 10 items that measure the degree of exposure to adverse physical factors and working conditions. Examples are “The temperature is high in my working environment” and “My work is dangerous”. The participants indicated their agreement with the statements on a 5-point Likert scale (1 = completely inconsistent, 5 = completely consistent). The ratings of the 10 items were averaged to form an indicator of the perception of working environmental risk.

Other Occupational Stressors: There are 6 dimensions in the *ORQ*. In addition to *PE*, the other five dimensions, which are *role overload (RO), role insufficiency (RI), role ambiguity (RA), role boundary (RB),* and *responsibility (R),* were measured as control variables. RO measures the extent to which job demands exceed employee capabilities and workplace resources and the extent to which the employee can meet the demands of their workload (10 items). RI measures how well an employee’s training, education level, skills, and experience fit the needs of the job (10 items). RA measures how clear an employee is about the focus of task, schedule, expectations, and evaluation criteria in work (10 items). RB measures conflicting task requirements and work commitment experienced by employees (10 items). R measures the degree to which an employee has or feels responsible for the performance and well-being of others (10 items). The participants indicated their agreement with the statements on a 5-point Likert scale (1 = completely inconsistent, 5 = completely consistent). The ratings in each dimension were averaged to form indicators of other occupational stressors.

Occupational Strain: The *Personal Strain Questionnaire (PSQ)* subscale of the *OSI-R* was used to measure the occupational strain of geological investigators. The PSQ consists of four dimensions, which are *vocational strain (VS), psychological strain (PSY), interpersonal strain (IS),* and *physical strain (PHS)*. VS measures the extent to which the employee has problems with work quality and output and also measures work attitude with 10 items, for example, “I am bored with my job”. PSY measures the extent to which an employee experiences psychological and emotional problems with 10 items, for example, “Lately, I get angry easily”. IS measures how disorganized an employee is in their relationships (e.g., withdrawal, aggressive behavior) with 10 items, for example, “I often get into arguments with people close to me”. PHS measures an employee’s chief complaints of physical disorders and poor self-care habits with 10 items, for example, “Lately, I have been feeling tired”. The participants indicated their agreement with the statements for the past month on a 5-point Likert scale (1 = completely inconsistent, 5 = completely consistent). The ratings in each dimension were averaged to form indicators of the dimensions of occupational strain.

Perceived Organizational Support: The *Perceived Organizational Support Questionnaire (POSQ)* by Chen etc. al. [44] was used to measure perceived organizational support. The questionnaire consists of 2 dimensions, which are *perceived socio-emotional support* and *instrumental support* from the organization. The socio-emotional support section consists of 7 items, for example, “My organization appreciates my contribution” and “My organization respects my goals and values”. The instrumental support section consists of 3 items, for example, “My organization tries its best to provide a good working environment and facilities” and “My organization tries its best to provide the necessary personnel and information support for work”. The participants indicated their agreement with the statements on a 5-point Likert scale (1 = strongly disagree, 5 = strongly agree). The reliability and validity of this questionnaire were tested in a previous study. The ratings in each dimension were averaged to form indicators of the perceived socio-emotional support and instrumental support.

### 2.4. Reliability and Validity of Questionnaires

The reliability and validity of the ORQ and PSQ from the OSI-R and the POSQ were tested with this sample (see Table 1). The Cronbach’s *α*s of most dimensions were greater than 0.7, and only two dimensions had a Cronbach’s *α* of less than 0.7, namely *α_RO_* = 0.65 and *α_IS_* = 0.69. According to Aiken’s (2008) criteria, a reliability coefficient of 0.60 to 0.70 can be satisfactory [45]. Therefore, the reliability of the questionnaires in this study can be accepted. Confirmatory factor analysis was carried out for each questionnaire to test the construct validity. The critical values of the fit indices are as follows: *TLI, CFI, GFI* > 0.9, *RMSEA* < 0.08 [46]. In this study, most of the fit indices met the requirements, indicating that the models are acceptable and the questionnaires have good construct validity. However, the *RMSEA* of the PSQ was 0.087, slightly higher than 0.08. Browne and Cudeck (1993) pointed out that an *RMSEA* > 1 is unacceptable [47]. Considering that the *TLI*, *CFI*, and *GFI* of the PSQ model were all greater than 0.9, the construct validity of the questionnaire is also considered acceptable.

## 3. Results

### 3.1. Common Method Biases

In this study, a self-reported questionnaire survey was adopted, so Harman’s single-factor test was used to evaluate the common method bias [38]. Exploration factor analysis was carried out on 60 items of independent variables, dependent variables, and potential moderators. (Note: The sample size in this study was not large enough to conduct exploratory factor analysis for all 110 questions. So, we had to take the second best and perform a common method bias test with the main variables (60 items in total) of the study, namely the variables excluding the control variables. We conducted regression analysis without the control variables. The results show that the relationships among the independent variables, potential moderators, and dependent variables were not different from those presented in the paper. That is, whether the control variables were included in the common method bias test did not affect the conclusion of this study.). The results show that 14 common factors with eigenvalues of more than 1 were extracted, and the variance interpretation rate of the first common factor was 25.75%, which is less than 40%. Therefore, there was no serious common method bias in this study.

### 3.2. Perception of Working Environmental Risk and Occupational Strain of Geological Investigators

First, in order to assess the working environmental risk perception and occupational strain of geological investigators, we performed descriptive statistics and compared them with the data of administrative staff from the same organization who do not work in the field. The results support Hypothesis 1; that is, the working environmental risk perception of geological investigators (*M* = 28.07, *SD* = 7.99) was higher than that of the administrative staff (*M* = 22.17, *SD* = 9.34, *t* = 4.791, *p* < 0.001). For the dimensions of occupational strain, psychological strain, interpersonal strain, and physical strain, the geological investigators had higher values than the administrative staff (see Table 1). Second, the OSI-R has multiple occupational norms in China [43]. We compared the working environmental risk perception and occupational strain of geological investigators with the norms of managers, scientific researchers, safety service personnel, and technical workers. The T-test showed that the working environmental risk perception of geological investigators (*M* = 28.07, *SD* = 7.99) was higher than that of managers (*M* = 22.23, *SD* = 6.63, *t* = 10.59, *p* < 0.001), scientific researchers (*M* = 25.48, *SD* = 7.13, *t* = 4.70, *p* < 0.001), and security service personnel (*M* = 24.51, *SD* = 7.00, *t* = 6.46, *p* < 0.001), and did not significantly differ from that of technical workers (*M* = 29.05, *SD* = 8.09, *t* = −1.77, *p* = 0.077). In terms of the occupational strain of geological investigators, all dimensions were at a high level compared to other occupations, except for interpersonal strain, for which the investigators experienced a medium level. The total occupational strain of geological investigators was also significantly higher than that of researchers and security service personnel (see Table 2).

### 3.3. The Effect of Working Environmental Risk Perception on Occupational Strain: Role of Perceived Organizational Support

In order to clarify the effect of geological investigators’ working environmental risk perception on occupational strain and the role of perceived organizational support, we conducted a hierarchical multiple regression analysis after centralizing all the variables.

Before the regression analysis, assumption tests were conducted [48]. Linearity and homoscedasticity assumptions were tested using the plots of unstandardized predicted values versus studentized residuals. The multicollinearity assumption was tested by the inflation factor (VIF) and tolerance values. The minimum tolerance value was 0.79 (>0.2) and the maximum VIF value was 1.27 (<10), indicating that the assumption was met. The influential cases assumption was tested using Cook’s distance. The maximum value was 0.08 (<1), indicating that there were no influential cases. A Q-Q plot showed that the residuals of the model were normally distributed.

First, regression was conducted with working environmental risk perception as an independent variable, occupational strain as a dependent variable, several demographic variables (including gender, working years, ranks, and months of field work per year), and other stressors as control variables. The results are consistent with Hypothesis 2, which indicates that working environmental risk perception significantly predicted occupational strain, even after controlling for demographic variables and other stressors, with the highest regression coefficient of all predictors (*β* = 0.34, *t* = 5.635, *p* < 0.001). This means that working environmental risk is an important source of occupational strain. The working years also negatively predicted occupational strain (*β* = −0.23, *t* = −2.515, *p* = 0.013).

Secondly, the two dimensions of perceived organizational support were taken as independent variables in the regression analysis. Perceived socio-emotional support (*β* = −0.22, *t* = −3.531, *p* = 0.001) and instrumental support (*β* = −0.13, *t* = −2.046, *p* = 0.042) both negatively predicted occupational strain.

Third, in order to test whether the two dimensions of perceived organizational support have a moderating effect, we multiplied working environmental risk perception with perceived socio-emotional support and perceived instrumental support. The interaction items were included in the regression (see Table 3). The results show that the prediction of socio-emotional support was still significant (*β* = −0.20, *t* = −3.086, *p* = 0.002), while the interaction of working environmental risk perception and perceived socio-emotional support was not a significant predictor (*β* = 0.04, *t* = 0.668, *p* = 0.505). This supports Hypothesis 3; that is, socio-emotional support is not a moderator. However, the main effect of perceived instrumental support was replaced by the interaction of working environmental risk perception and instrumental support (*β* = −0.18, *t* = −3.268, *p* = 0.001). Instrumental support could not significantly predict occupational strain (*β* = −0.11, *t* = −1.842, *p* = 0.067). This supports Hypothesis 4; that is, perceived instrumental support moderates the relationship between working environmental risk perception and occupational strain.

**Figure 1:**
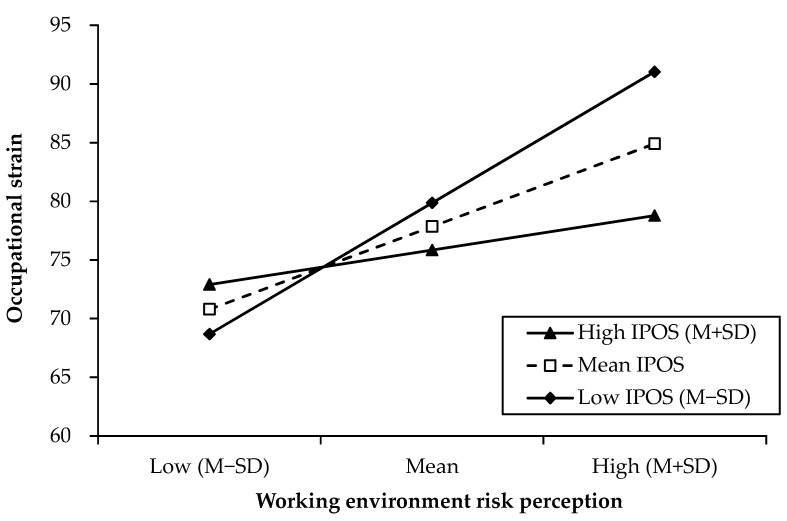
Simple slopes of perceived instrumental support in working environmental risk perception on occupational strain.

A simple slope test was conducted to further illustrate the moderating effect of perceived instrumental support. The simple slopes of the three groups of high, mean, and low perceived instrumental support are shown in Table 4 and Figure 1. The results show that for participants with low (*b* = 1.55, *t* = 6.491, *p* < 0.001) and mean (*b* = 0.91, *t* = 5.660, *p* < 0.001) perceived instrumental support, working environmental risk perception significantly predicted occupational strain. However, for participants with high perceived instrumental support, working environmental risk perception was no longer significant in predicting occupational strain (*b* = 0.28, *t* = 1.064, *p* = 0.289). That is, perceived instrumental support can effectively buffer the stress consequences caused by working environmental risks.

## 4. Discussion

### 4.1. Environmental Risks and Occupational Strain of Geological Investigators

There are risks in geological survey environments, and thus, geological investigators should have a strong sensitivity to risks to avoid accidents. However, the perception of risk can cause stress, resulting in adverse work, physical, and psychological outcomes. This study explores how the negative impact of working environmental risk on mental health can be reduced without losing risk sensitivity through perceived organizational support.

The first issue this study looked at was whether the working environmental risk and occupational strain of geological investigators are at a high level. In response to this problem, the study adopted the OSI-R, a standardized questionnaire package with many occupational norms in China. Using these questionnaires for measurement, geological investigators could be compared to control groups to clarify the level of working environmental risk and occupational strain. First, the results support Hypothesis 1: For geological investigators working in a high-risk environment, they scored higher on working environmental risk perception than administrative staff from the same organization, and Chinese managers, scientific researchers, and security service personnel. However, their scores were not significantly different from the scores of the technical workers. Technical workers refer to workers engaged in front-line work in production and manufacturing who are exposed to adverse environments or risks in production workshops, while geological investigators are exposed to adverse environments or risks in the field. There are many risk factors in both working environments. Second, the total occupational strain of geological investigators was not significantly different from that of managers and technical workers, but higher than that of administrative staff, scientific researchers, and security service personnel. Geological investigators were also at a high or medium level in each subdimension of occupational strain. The psychological strain of geological investigators was especially high and greater than that of all the comparative groups in this study. This dimension is a key psychological factor of occupational strain [49], reflecting an individual’s emotional response to stress. Participants with high scores are depressed, anxious, and irritable. The vocational strain and physical strain of geological investigators were also high, which are related to a negative attitude, low interest, and a lack of concentration at work, and somatization such as pain, fatigue, and back pain. It is also associated with negative outcomes, such as accidents and absences from work.

To explain the impact of working environmental risk on occupational strain, the total score of the strain dimensions was used as the dependent variable for subsequent regression analysis. The results support Hypothesis 2: after controlling for demographic variables and other occupational stressors, the predictive effect of working environmental risk on occupational strain is still significant. In addition to working environmental risk, role insufficiency, role boundary, and responsibility were significant predictors of occupational strain, while role overload and role ambiguity could not predict the strain of geological investigators. This is not completely consistent with the results of a study that conducted a meta-analysis on the relationship between stressors and stress responses, which showed that role ambiguity (*ρ* = −0.24) was more closely correlated with job performance than environmental uncertainty (*ρ* = −0.11) and job insecurity (*ρ* = 0.19) [50]. The reason for this discrepancy may be due to the characteristics of geological surveys. The work and schedule of the field geological investigators are relatively clear. There are very few cases of ambiguity or urgent work assignments. However, geological investigators may experience occupational strain because they feel that their skills are not sufficient to meet the needs of the job or that their work progress is slow, among other things.

This study also responded to the debate on the stress conservation of resources theory and the adaptation theory; that is, whether strain increases or decreases with continuous working environmental risks. The results suggest that occupational strain decreases with longer working years, which supports the adaptive model of stress [51]. After adding perceived organizational support to the regression analysis, the prediction effect was marginally significant (*β* = −0.16, *p* = 0.063 in Model 2; *β* = −0.14, *p* = 0.088 in Model 3). The results indicate that increasing perceived organizational support as time passes may partly explain the effect of working years on occupational strain. However, in general, the negative prediction trend still exists; that is, with increasing working years, geological investigators gradually adapt to environmental risks and the influence of stressors weakens.

### 4.2. Effect of Perceived Socio-emotional and Instrumental Support

Perceived organizational support refers to the general perception of employees that the organization values their contribution and cares about their welfare [21]. Traditional organizational support focuses only on intimacy and respect, which is characterized by social and emotional support. However, in addition to emotional support, organizations can provide employees with instrumental support, including information, tools, training, and other factors. In this study, organizational support was divided into socio-emotional and instrumental support to explore whether these two types of organizational support play the same role in occupational strain. An important finding is that the geological investigators’ perceived socio-emotional and instrumental support had differential mechanism patterns.

The results of this study suggest that perceived socio-emotional support has a moderate effect on occupational strain, which parallels that of environmental stressors and can counteract the stress response caused by environmental stressors. Individualized consideration from the organization, mutual assistance, and intimacy between employees and leaders and other members can provide socio-emotional support [52]. From the perspective of conservation of resources theory, managers’ concern for employees’ welfare, respect for employees, and recognition of their value in the organization may be helpful in reducing work–family conflicts, providing a perception of social support, and enabling employees to gain resources (such as enhanced self-esteem). These benefits can help workers cope with stress and improve employee performance [53].

Perceived instrumental support plays a moderating role by influencing the relationship between working environmental risk perception and occupational strain, rather than directly affecting the occupational strain. The lower the instrumental support perceived, the closer the relationship between working environment risk perception and occupational strain. Perceived instrumental support comes from the organization providing the necessary information, equipment, material, and people support to employees. For geological investigators with low perceived instrumental support, working environmental risks have a greater negative impact. With increasing environmental risk, strain also increases. The reason for this is that without instrumental support, geological investigators can only rely on themselves to cope with the risks of the working environment. However, in the case of high perceived instrumental support, occupational strain scores did not exhibit a significant difference between high and low working environmental risk perception. The reason for this is that perceived instrumental support provides an objective means for geological investigators to deal with environmental stressors. Even if there are risks in the working environment, they still feel that they have practical methods they can use to reduce the risk and sufficient confidence in risky situations. Additionally, environmental risk went from a hindrance stressor to a challenge stressor [37]. Instrumental support may also provide employees with coping resources by improving their competence and efficacy [36]. Thus, it can reduce the impact of stressors on physical and mental health. This suggests that an organization providing instrumental support can mitigate the negative effects of environmental stressors without reducing workers’ situational awareness.

### 4.3. Limitations and Suggestions for Future Research

Firstly, the measurements of variables such as working environmental risk perception and perceived organizational support were self-reported and may have been affected by individual characteristics. Different individuals may have different feelings towards the same working environment and the same organizational management system. Based on the perspective of conservation of resource theory, this study assumed that stressors are the determinants of stress response and the perception of the same stressor is generally similar across individuals. However, stress appraisal theory and other theories challenge this, proposing that an individual’s perception of stressful events is the decisive factor of stress. Experiencing the same event can be stressful for some but not for others [54,55]. Therefore, in future studies, individual differences in working environmental risk perception and perceived organizational support should be included in the research.

Secondly, the relationships between different dimensions of occupational strain and working environmental risk perception were slightly different. To explain the general relationship and due to space limitations, the scores of four dimensions of occupational strain were combined into the total strain index and analyzed as one dependent variable. In fact, working environmental risk perception had a higher correlation with physical strain (*r* = 0.54 ***) than with vocational strain (*r* = 0.37 ***). Additionally, the correlations between perceived organizational support and the dimensions of occupational strain were also complex. These subtle differences may have theoretical implications. In future studies, we could explore the relationships between working environmental risk perception and different dimensions of occupational strain to better understand the mechanism and intervene.

Thirdly, a questionnaire survey was used in this study, and while the results obtained can illustrate the correlation between variables, they cannot infer causality. In future studies, more research methods should be adopted, such as comparison at the organizational level in different geological survey institutions, or intervention studies. In these ways, clearer relationships between working environmental risk, organizational support, and occupational strain can be illustrated.

## 5. Conclusions

The perception of working environmental risk and occupational strain of geological investigators was found to be higher than that of administrative staff, managers, and researchers. Working environmental risk is one of the most prominent stressors for geological investigators. Different dimensions of perceived organizational support play different roles in coping with stress; socio-emotional support directly reduces occupational strain, while instrumental support buffers the effect of risk perception on occupational strain.

## Figures and Tables

**Table 1:** Reliability and validity of questionnaires (*N* = 268).

	Number of Items	Number of Dimensions	Reliability	Validity				
			Cronbach’s *α*	*χ* ^2^ */df*	*RMSEA*	*TLI*	*CFI*	*GFI*
*Occupational Role Questionnaire (ORQ)*	60	6	PE: 0.87; RO: 0.65; RI: 0.81;RA: 0.75; RB:0.73; R:0.81; Total: 0.89	2.088	0.064	0.962	0.995	0.995
*Personal Strain Questionnaire (PSQ)*	40	4	VS: 0.82; PSY: 0.87; IS: 0.69; PHS: 0.86; Total: 0.94	3.027	0.087	0.980	0.997	0.994
*Perceived Organizational Support Questionnaire (POSQ)*	10	2	Socio-emotional POS: 0.80; Instrumental POS: 0.80; Total: 0.87	1.968	0.060	0.954	0.966	0.953

**Table 2:** Means, standard deviations, and T-tests of working environmental risk perception and occupational strain.

	Geological Investigators (*n* = 210)	Administrative Staff (*n* = 58)		Managers (*n* = 569)		Scientific Researchers (*n* = 235)		Security Service Personnel (*n* = 331)		Technical Workers (*n* = 9903)	
	*M ± SD*	*M ± SD*	*t*	*M ± SD*	*t*	*M ± SD*	*t*	*M ± SD*	*t*	*M ± SD*	*t*
Working environmental risk perception	28.07 ± 7.99	22.17 ± 9.34	4.791 ***	22.23 ± 6.63	10.59 ***	25.48 ± 7.13	4.70 ***	24.51 ± 7.00	6.46 ***	29.05 ± 8.09	−1.77
Occupational strain											
Vocational strain	21.72 ± 6.18	19.86 ± 7.47	1.932	22.54 ± 3.14	−1.92	20.35 ± 5.38	3.21 **	20.03 ± 5.66	3.96 ***	22.57 ± 3.74	−1.99 *
Psychological strain	25.97 ± 7.71	23.43 ± 7.10	2.258 *	24.27 ± 4.88	3.197 **	23.86 ± 6.06	3.97 ***	24.42 ± 6.84	2.92 **	24.10 ± 5.29	3.52 ***
Interpersonal strain	26.50 ± 5.12	24.91 ± 5.58	2.053 *	27.30 ± 3.41	−2.25 *	25.66 ± 4.21	2.39 *	24.37 ± 4.77	6.04 ***	27.46 ± 3.76	−2.70 **
Physical strain	24.29 ± 7.43	19.84 ± 6.93	4.093 ***	22.36 ± 4.02	3.766 ***	21.29 ± 5.37	5.85 ***	24.49 ± 6.91	−0.39	23.34 ± 4.73	1.85
Total strain	98.49 ± 22.88	88.05 ± 23.44	3.058 **	96.48 ± 11.81	1.27	91.15 ± 18.11	4.65 ***	93.31 ± 20.30	3.28 ***	97.47 ± 13.77	0.64

* *p* < 0.05, ** *p* < 0.01, *** *p* < 0.001. Managers include administrative business managers and administrative affairs managers. Scientific researchers include those from the literature, science, sociology, education, and other disciplines. Security service personnel include police, security personnel, and firefighters. Technical workers include personnel working in the workshops of chemical plants, manufacturing factories, electronic factories, pharmaceutical factories, and printing factories, as well as power equipment operation, maintenance, power supply workers, etc.

**Table 3:** Hierarchical multiple regression predicting occupational strain from working environmental risk perception and perceived organizational support.

Predictor	Model 1 (Independent Variables and Control Variables)			Model 2 (Potential Moderators)			Model 3 (Interactions)		
	*β*	*t*	*p*	*β*	*t*	*p*	*β*	*t*	*p*
Demographic									
Gender	−0.03	−0.643	0.521	−0.076	−1.546	0.124	−0.08	−1.604	0.111
Working years	−0.23	−2.515	0.013	−0.16	−1.869	0.063	−0.14	−1.715	0.088
Ranks	0.09	0.959	0.339	0.04	0.634	0.527	0.02	0.188	0.851
Months of field work per year	0.01	0.150	0.881	−0.01	−0.295	0.769	−0.02	−0.407	0.685
WERP	0.34	5.635	0.000	0.32	5.797	0.000	0.30	5.319	0.000
Other stressors									
Role overload	0.06	0.928	0.355	0.05	0.845	0.399	0.05	0.859	0.392
Role insufficiency	0.23	2.935	0.004	0.18	2.509	0.013	0.18	2.616	0.010
Role ambiguity	0.03	0.337	0.737	0.04	0.530	0.597	0.03	0.464	0.643
Role boundary	0.29	4.122	0.000	0.20	3.061	0.003	0.20	3.145	0.002
Responsibility	0.15	2.269	0.025	0.11	1.820	0.071	0.12	2.030	0.044
POS									
Socio-emotional POS				−0.22	−3.531	0.001	−0.20	−3.086	0.002
Instrumental POS				−0.13	−2.046	0.042	−0.11	−1.842	0.067
WERP × Socio-emotional POS							0.04	0.668	0.505
WERP × Instrumental POS							−0.18	−3.268	0.001
***R* ^2^**	0.560			0.638			0.662		
**△** ***R* ^2^**	0.551			0.078			0.024		
***F***	*F*(10,171) = 21.728, *p* < 0.001			*F* (12,169)= 24.844, *p* < 0.001			*F* (14,167)= 23.330, *p* < 0.001		

WERP = working environmental risk perception, POS = perceived organizational support. Ranks are divided into junior, intermediate, and senior.

**Table 4:** Simple slope test of perceived instrumental support in working environmental risk perception and occupational strain.

Level of Moderator	*b*	*SE*	*t*	*p*	*95% CI*	
High Instrumental POS(+1*SD*)	0.28	0.26	1.064	0.289	−0.24	0.79
Mean Instrumental POS	0.91	0.16	5.660	0.000	0.59	1.23
Low Instrumental POS(−1*SD*)	1.55	0.24	6.491	0.000	1.08	2.02

Control variables: demographic variables (including gender, working years, ranks, and months of field work per year), other stressors (role overload, role insufficiency, role ambiguity, role boundary, responsibility) and perceived socio-emotional support.

## Data Availability

The data supporting the reported results can be found at https://pan.baidu.com/s/1gTTQ4v0fo78eZ24f5-oKfw?pwd=ims3, code: ims3. (accessed on 17 November 2022).
